# Undergraduate dental students’ perspective on the implementation of digital dentistry in the preclinical curriculum: a questionnaire survey

**DOI:** 10.1186/s12903-020-01071-0

**Published:** 2020-03-18

**Authors:** Maximiliane Amelie Schlenz, Karin Michel, Kerstin Wegner, Alexander Schmidt, Peter Rehmann, Bernd Wöstmann

**Affiliations:** Dental Clinic - Department of Prosthodontics, Justus Liebig University, Schlangenzahl 14, 35392 Giessen, Germany

**Keywords:** Dentistry, Undergraduate medical education, CAD-CAM, Tooth preparation, Curriculum, Questionnaires, Dental students

## Abstract

**Background:**

Digitalisation is an expanding field in dentistry and implementation of digital teaching methods in dental education is an essential part of modern education. Therefore, two digital training modules were implemented in the preclinical curriculum at the Justus Liebig University Giessen. The aim of this study was to assess the students’ perspective on the implementation with a questionnaire survey.

**Methods:**

Since the fall term 2017/18, students of the course of dental prosthodontics I attended the training module I, where they learned to use computer-aided learning (CAL) approaches for the digital analysis of tooth preparations. In training module II, students of the course of dental prosthodontics II learned how to manufacture a computer-aided design/computer-aided manufacturing restoration. After the completion of the training modules, all students starting with the fall term 2017/18 to the spring term 2019 were asked to fill in a questionnaire regarding the aspects of handling, didactic benefit, motivation, and overall assessment.

**Results:**

Students rated the implementation of digital aspects in teaching as positive in terms of handling, didactic benefit, and motivation, but gave preference to the assessment of the tooth preparations by dental instructors. In addition, students assessed the feedback from the faculty regarding tips and tricks better than the digital feedback. More than 90% of the students indicated that they could imagine using an intraoral scanner for treatment of patients in the dental office in future.

**Conclusions:**

The results of the present study revealed a positive perspective of students on the implementation of digital dentistry in the preclinical curriculum. However, difficulties with CAL systems were reported and most students preferred evaluation of preparation by dental instructors. Thus, CAL approaches offer an additional teaching method besides the traditional teaching of manual skills.

## Background

Currently, digitalisation is an expanding field in dentistry, especially in terms of computer-aided design/computer-aided manufacturing (CAD/CAM) of dental restorations and appliances. For the fixed dental prostheses (FDPs), traditional manufacturing requires numerous manual steps by the dentist as well as by the dental laboratory. This process can be facilitated decisively using CAD/CAM technologies [1, 2]. Instead of conventional impressions with subsequent fabrication of plaster models, intraoral scanners can be used to digitise the intraoral situation. Subsequently, the restoration can be designed with CAD software and milled by computerised numerical control (CNC) machines. If necessary, models can also be fabricated using a CAD/CAM process. Due to the industrial manufacturing process, a standardised quality of the FDPs can be ensured and the production time with overall costs are reduced [2]. However, as for all technical innovations, high-quality training of users is necessary for successful implementation of CAD/CAM technologies in daily patient care [1, 3]. Thus, implementation of digital techniques and workflows is indispensable in contemporary education of dental students. However, publications in this field mostly reflect on the performance of CAL systems [3–8], whereas the view of the students is only sparsely analysed. Thus, this study is primarily focussed on the students’ perspective.

In most universities, education of dental students is still solely focused on conventional teaching methods including traditional laboratory techniques such as waxing, casting, finishing, and tooth preparation exercises on the phantom head (simulation unit). Thus, the challenge was to implement new digital technologies such as the so-called computer-aided learning (CAL), without neglecting the training of manual skills, which are still important for dental treatment [5–7, 9, 10].

Since the fall term 2017/18, students of the Justus Liebig University Giessen were introduced to digital technologies in the preclinical curriculum. Students still perform their exercises on the phantom heads, but they also use the CAL software for additional support. Especially for students who prepare a tooth for the first time, it is difficult to understand and fulfil the preparation requirements. *Knight* suggested that it is important to have a precise idea of the final result for the training of manual skills [11]. Currently, CAL approaches can support traditional teaching methods [8]. Furthermore, students’ awareness for tooth structure loss can be increased by using three-dimensional imaging of their own preparations and by superimposition of the images on the original tooth in the CAL software for the self-assessment process. This is especially important, as studies have shown that self-assessment increases the motivation to learn and supports lifelong learning [5, 12].

Students need an individual feedback on their performance to learn from their mistakes and to get a sense of their accomplishments [11]. Therefore, a high reliability of the evaluation process is essential. However, studies regarding the traditional evaluation of preparations according to the glance-and-grade principle have reported a low interrater (between different examiners) and intrarater (one examiner at different times) reliability [5, 13, 14]. Furthermore, students request an immediate feedback with objective assessment criteria and suggestions for improvement [12, 15]. Due to different staff member opinions, work experiences, and evaluation standards as well as lack of time, it is often difficult to provide immediate feedback for each student in everyday teaching. This may entail a lack of objectivity and disagreement between students and staff members [5, 12]. *Renne* et al. reported that students often perceive assessments as subjective and arbitrary [16]. This perception may lead to decreased self-awareness and may negatively influence students’ performance [17]. Taken together, at the moment each university seems to develop an individual concept. General approaches – even country based – could not be identified in the literature.

Digital methods such as CAL are especially suitable in such situations, as they provide an objective and immediate feedback. They allow for an individual learning speed and are available when required by the students [1, 3, 6, 7, 9, 18]. Thus, the workload of the faculty staff is reduced in the long term [5]. However, such methods require high investment costs at the beginning and their implementation in the existing curriculum is time-consuming [6, 19, 20]..

At the Justus Liebig University Giessen, two training modules were implemented in the preclinical education. These modules consisted of students learning to analyse their preparations digitally (I) and to accomplish a complete CAD/CAM workflow for FDPs (II). Nevertheless, for the success of an educational concept, it is important to consider the students’ perspective [5]. Therefore, the aim of this study was to evaluate students’ opinions about these training modules through questionnaires.

## Methods

### Teaching concept

At the Justus Liebig University Giessen, faculties (dentists) of the Department of Prosthodontics supervise (MAS, KM, KW and AS) two classes of the preclinical curriculum: *course of dental prosthodontics I* (Ph1) in the third semester and *course of dental prosthodontics II* (Ph2) in the fifth semester. Students are allowed to use the preclinical laboratory during free practicing hours besides the official course lessons. The aim of the preclinical education is to learn the theoretical and the practical basics of prosthodontics, which students can apply to patients in the clinical curriculum later. This includes learning the correct preparation techniques for different types of FDPs and the complete workflow from preparation to impression making and manufacturing.

**Fig. 1:**
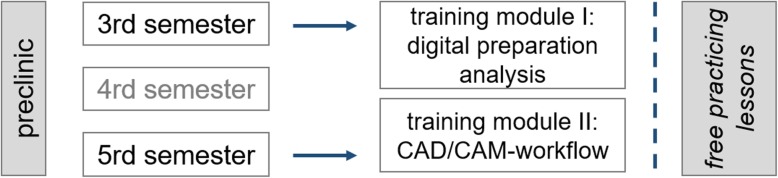
Teaching concept

To implement digital dentistry in the existing curriculum, training modules for theoretical and practical knowledge transfer were implemented in both the preclinical prosthodontic courses since the fall term 2017/18 (Fig. 1). Students also had the opportunity to use a digital preparation analysis software (prepCheck, Dentsply Sirona, Bensheim, Germany) by themselves during the free practicing lessons. Thus, the preparation analysis was digitised and the digital workflow of the CAD/CAM manufactured FDPs was already taught in the preclinical curriculum.

### Training module I – digital preparation analysis

Students of the third semester (18 male, 73 female, 13 not specified) were introduced to the digital preparation analysis during a 45-min lecture. The main targets of preparation exercises such as training of manual skills and self-assessment were repeated and basic information on digital preparation analysis was provided. Subsequently, students watched a step-by-step video of prepCheck. The application of the software with intraoral scanning was demonstrated on the phantom head in small groups of a maximum of five students. In addition, students received a handout with all the steps of the preparation analysis and a guide for the interpretation of the prepCheck report.

In order to give students the opportunity to compare their own preparations to preset parameters and to the so-called *master preparation*, outstanding preparations of students from the preliminary dental exam of the summer semester 2017 were stored in the software.

**Fig. 2:**
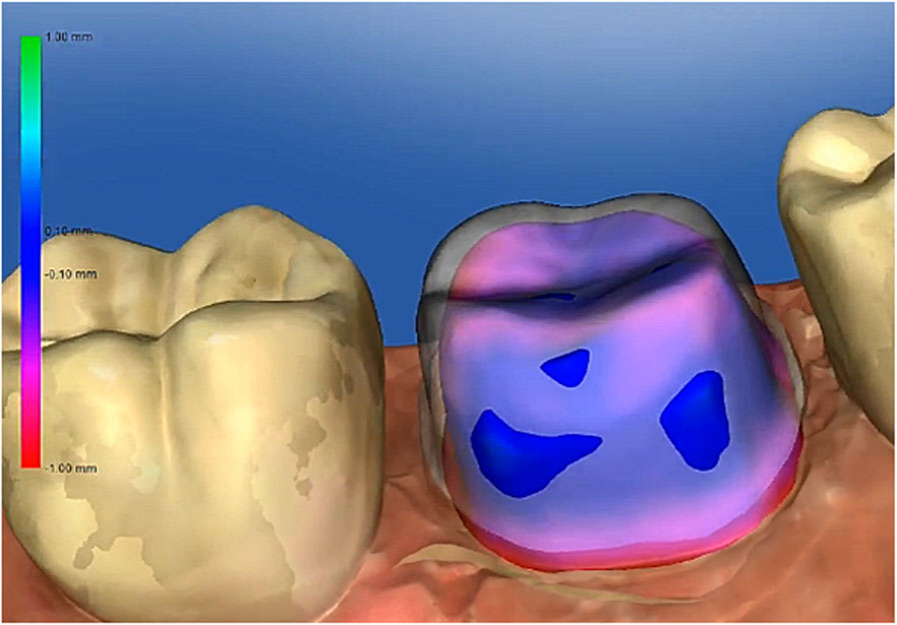
Digital preparation analysis with master preparation (colored = student preparation, white = master preparation)

**Fig. 3:**
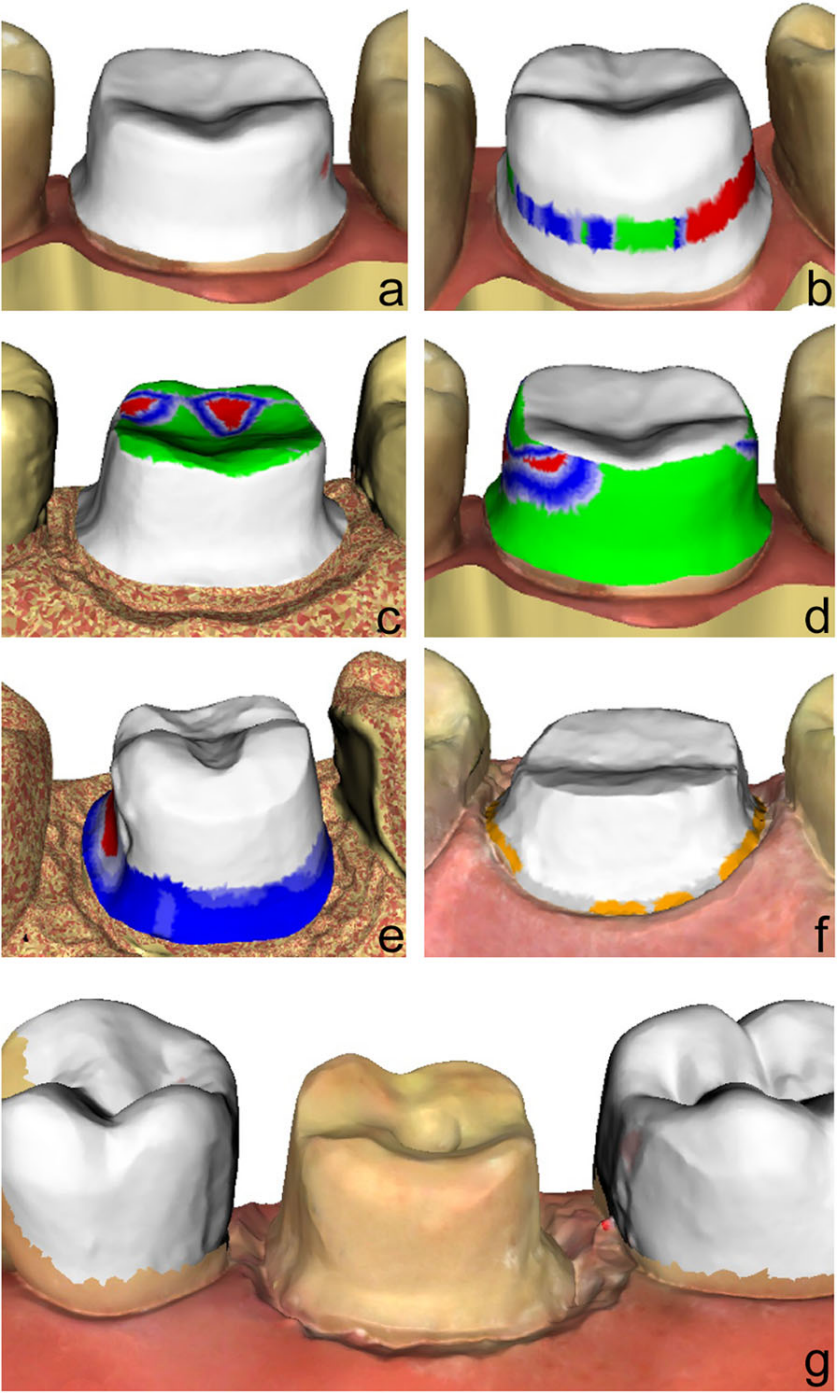
Digital preparation analysis with preset parameters (**a** = undercuts, **b** = preparation angle, **c** = occlusal reduction, **d** = axial reduction, **e** = preparation of margin line, **f** = surface of margin line, **g** = integrity of adjacent teeth)

The steps for the digital preparation analysis were as follows:

1. Intraoral scan: To perform the digital preparation analysis with prepCheck, students selected the tooth and the type of restoration (full crown in the present study) and scanned the preparation with the adjacent teeth, the antagonists, and the bite with the intraoral scanner (CEREC Omnicam, Dentsply Sirona, Bensheim, Germany). Subsequently, the model axis, the preparation margin, and the crown axis were defined. To analyse the preparation, the scanned data were imported into the prepCheck software.
2. Digital preparation analysis with master preparation: Students compared their preparations with the master preparation stored in the software (Fig. 2). The transparency of the three-dimensional student and master preparations could be modified using sliders. A colour scale on the student preparations showed the discrepancies between the two preparations. The deviations in millimetres were displayed in a legend.
3. Digital preparation analysis with parameters: Students compared their preparation to preset parameters regarding undercuts, preparation angle, occlusal and axial reduction, preparation margin, edges, surface texture, and the integrity of the adjacent teeth. Figure 3 shows an example of a student’s preparation analysed according to the different parameters.
4. PrepCheck report: The prepCheck analyses were exported in the ‘prepCheck report’ as a portable document format file, which was saved on the preclinical computers. They were also given to the students on a universal serial bus stick. Students presented their prepCheck reports in groups of two to train their self-assessment skills. Subsequently, the listening student gave feedback on the evaluation according to the sandwich principle [21].

All students analysed a full crown preparation of an upper incisor and a lower molar (21 and 46 according to the Fédération Dentaire Internationale [FDI] nomenclature), which they had already prepared in the running course. During the two-day training module, faculties were available to answer questions and help students with the CAL system. Whenever the digital preparation analysis showed need for improvement in the preparations, students were encouraged to correct their preparations and analyse the results with prepCheck again.

### Training module II – CAD/CAM workflow

An intraoral scanner (CEREC Omnicam) and a CNC milling machine (MC XL, Dentsply Sirona, Germany) were used for manufacturing the FDPs.

During the two-day training module, students of the fifth semester (25 male, 68 female, 4 not specified) were taught how to fabricate a crown using a CAD/CAM workflow. The first day started with a 60-min lecture giving students an overview of the CAD/CAM technology and a revision of the digital preparation analysis with prepCheck. Students also learned the theoretical background of digital impression making. Finally, the application of the CEREC software and the CNC milling machine were presented in a 20-min demonstration in small groups of a maximum of five students. During the remaining course time, students started with the CAD/CAM workflow for producing a posterior crown (FDI 46).

On the second day, a 45-min lecture was presented on CAD/CAM materials and their handling requirements. Subsequently, students were given a 20-min demonstration in small groups of a maximum of five participants to show them how to check the accuracy of fit and polish the ceramic (Celtra Duo, Dentsply Sirona, Germany). The remaining course time was used to finish the CAD/CAM crown. In addition to the lectures and the demonstrations, students received a script with background information regarding the CAD/CAM workflow.

The steps for the application of the CAD/CAM workflow were as follows:

1. Intraoral scan: Students created a fictional patient in the CEREC software. After selecting the tooth, restoration type (full crown), and material (Celtra Duo), an intraoral scan of the preparation including adjacent teeth, the antagonists, and the bite was performed with the Omnicam. Subsequently, the model axis, the preparation margin, and the crown axis were defined.
2. Digital preparation analysis: An objective preparation analysis with prepCheck was performed to analyse the preparation. The preparation was corrected and scanned for a second time if necessary.
3. Computer-aided design: Digital modelling of the crown was performed in the CEREC software. Initially, the software proposed a model, which could be modified using different sliders. It was possible to apply or remove substance and smoothen the restoration surface with a ‘digital wax knife’. The material-specific minimum layer thickness was also displayed. The occlusal and the proximal contact points could also be faded in and out. The objective was that the students should present a crown model with correct contact points, a sufficient minimum layer thickness, and appropriate shape.
4. Computer-aided manufacturing: A ceramic CAD/CAM block was inserted in the milling machine. Data were transferred from the Omnicam to the milling unit via wireless local area network connection. After milling, the restoration was carefully removed. The sprue was cut off with a diamond grinding wheel at 8000–12,000 rpm and polished with a rubber wheel.
5. Checking the fit: The space between the prepared tooth and the restoration was simulated with a low-viscosity silicone (Fit Test C&B, Voco, Cuxhaven, Germany). The strength of the occlusal and the proximal contact points was checked with occlusion foil (Hanel, Coltene, Altstätten, Switzerland) and dental floss.
6. Finishing and polishing: The contact points were corrected with a diamond bur if necessary. Crowns were finished with a diamond polishing body (< 60 μm) under light pressure at 8000–12,000 rpm, ensuring that the restoration did not overheat, followed by high-gloss polishing with a Robinson brush wheel and polishing paste (Zirkopol, feguramed GmbH, Buchen, Germany) at 4000–8000 rpm.

### Questionnaire survey

**Fig. 4:**
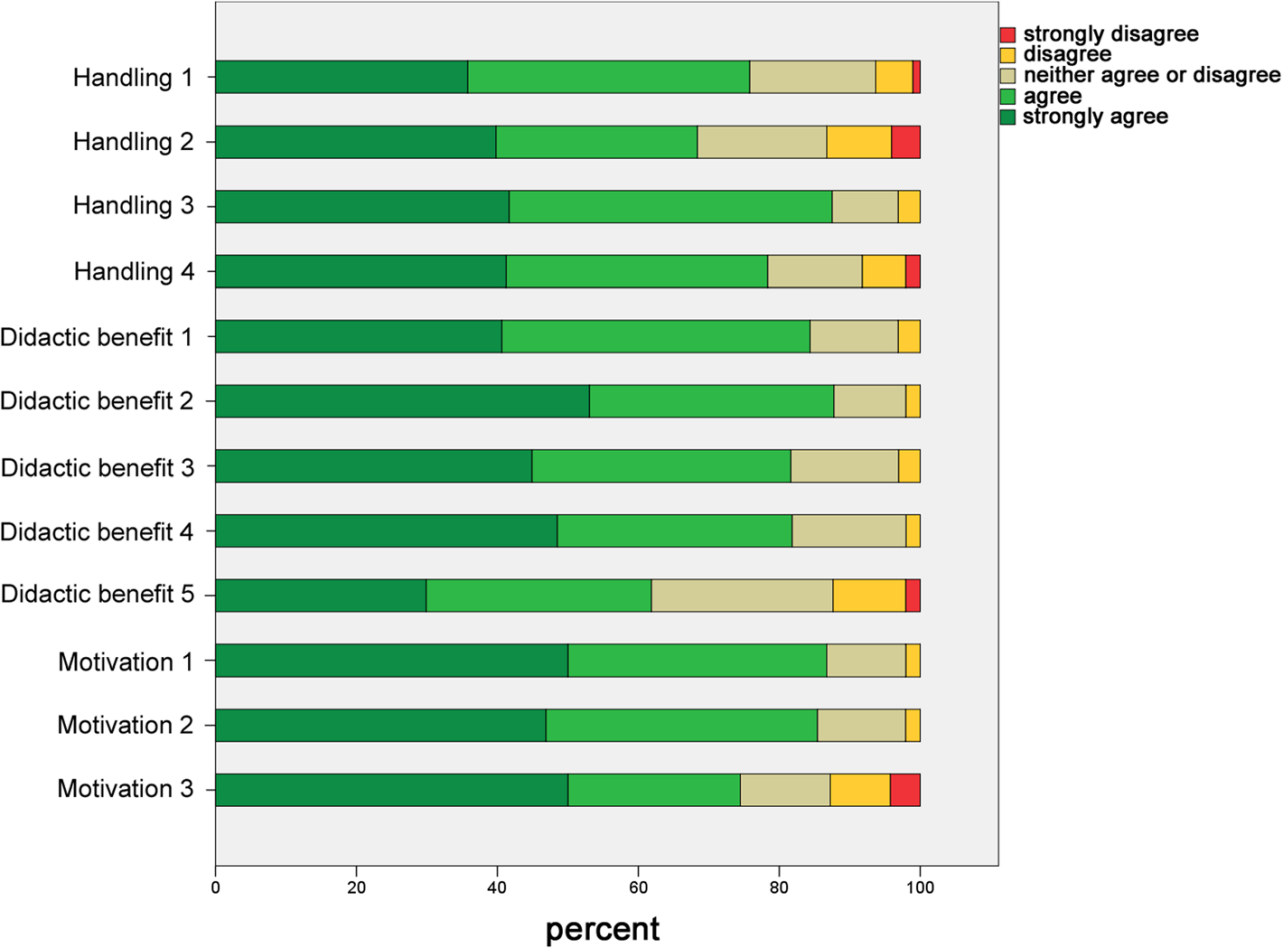
Depiction of the results of the questionnaire I – Digital preparation analysis regarding the aspects handling, didactic benefit and motivation

Two paper-and-pencile questionnaires (questionnaire I regarding the digital preparation analysis (Ph1) and questionnaire II regarding the CAD/CAM workflow (Ph2) were designed in cooperation with the Teaching Evaluation Service Centre of the Justus Liebig University Giessen. The questionnaires contained evaluative statements regarding the handling, didactic benefit, motivation, and overall assessment of digital dentistry. Students could agree or disagree with the statements based on a five-point Likert scale (Fig. 4) [22]. In addition, students were asked whether they preferred the assessment of the preparations by the software or by a dentist (Ph1). They were also asked if they preferred using conventional or digital workflow for manufacturing the FDPs (Ph2). At the end of the questionnaires, students were asked to answer a binary question (yes/no) if they could imagine using digital applications in their dental office in future. An abstention was allowed for each statement and the questionnaires were evaluated anonymously. All students of the course of dental prosthodontics I in the third semester and the course of dental prosthodontics II (Ph2) in the fifth semester between the fall term 2017/18 and the spring term 2019 participated in this study. Questionnaires were distributed at the end of the training modules and collected anonymously. The study was approved by the local ethics committee of the Justus Liebig University Giessen (Ref. no. 171/19).

Statistical analysis was performed using SPSS Statistics (version 24, IBM, Armonk, NY, USA). Median and percentiles were used to describe the data.

## Results

All students (*n* = 104) from the training module I (digital preparation analysis) and all students (*n* = 97) from the training module II (CAD/CAM workflow) answered the questionnaires. Nevertheless, due to the possibility of abstention, the number of valid answers per question ranged between 84.6 to 95.2% and 84.5 to 97.9% for modules I and II, respectively.

**Fig. 5:**
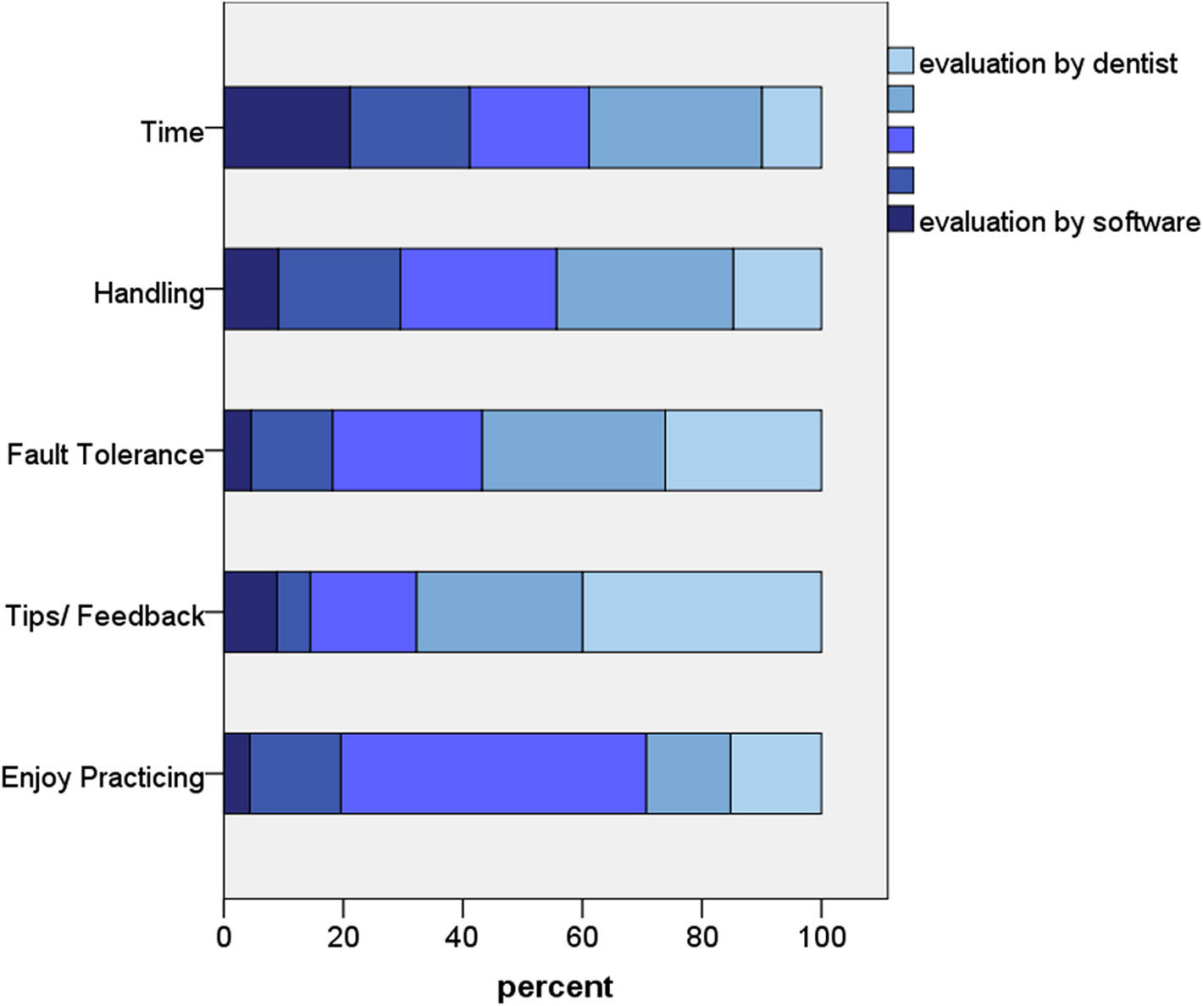
Depiction of the results of the questionnaire I – Digital preparation analysis regarding the aspects evaluation by dentist or software

**Fig. 6:**
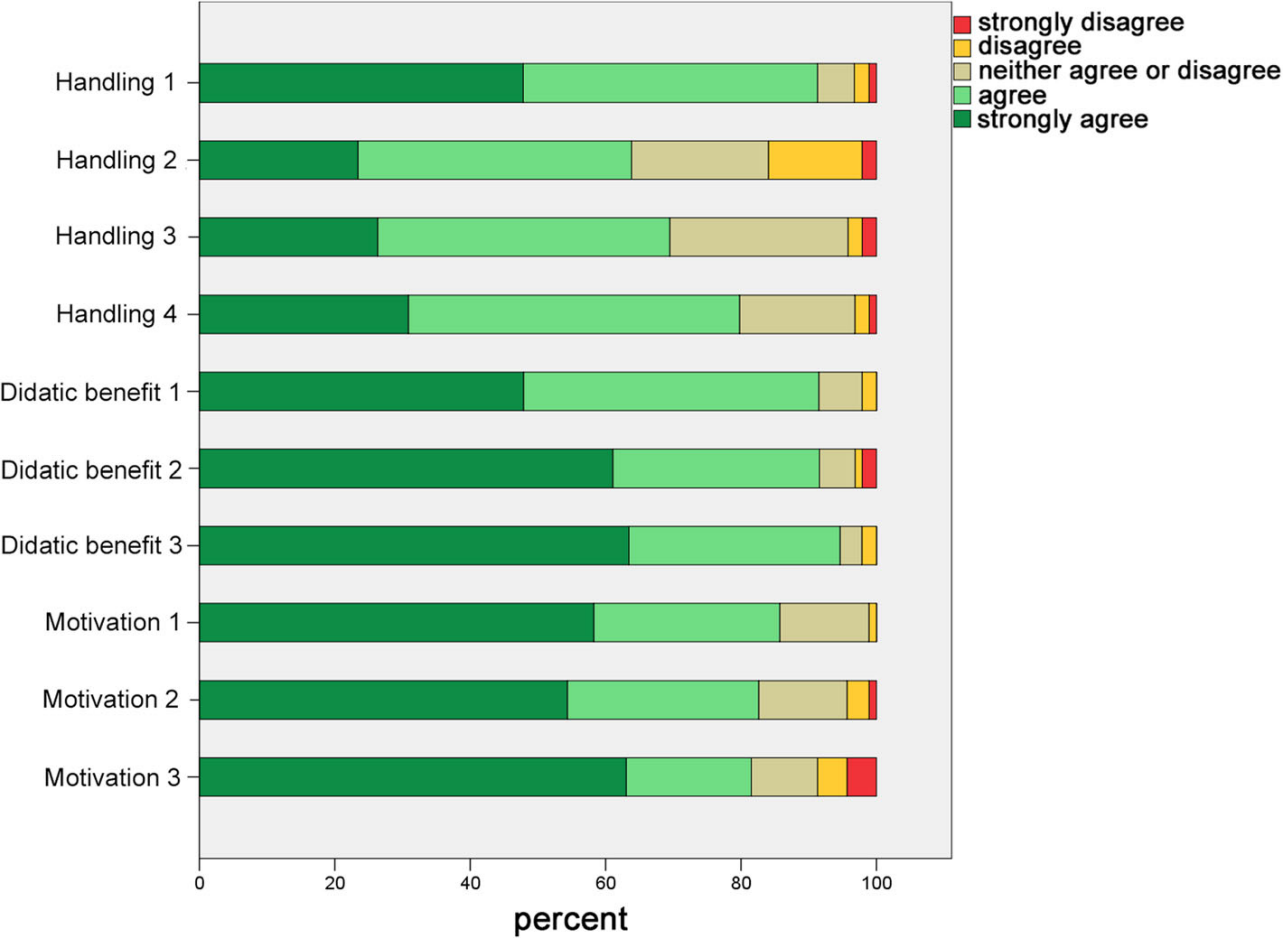
Depiction of the results of the questionnaire II – CAD/CAM-workflow regarding the aspects handling, didactic benefit and motivation

**Fig. 7:**
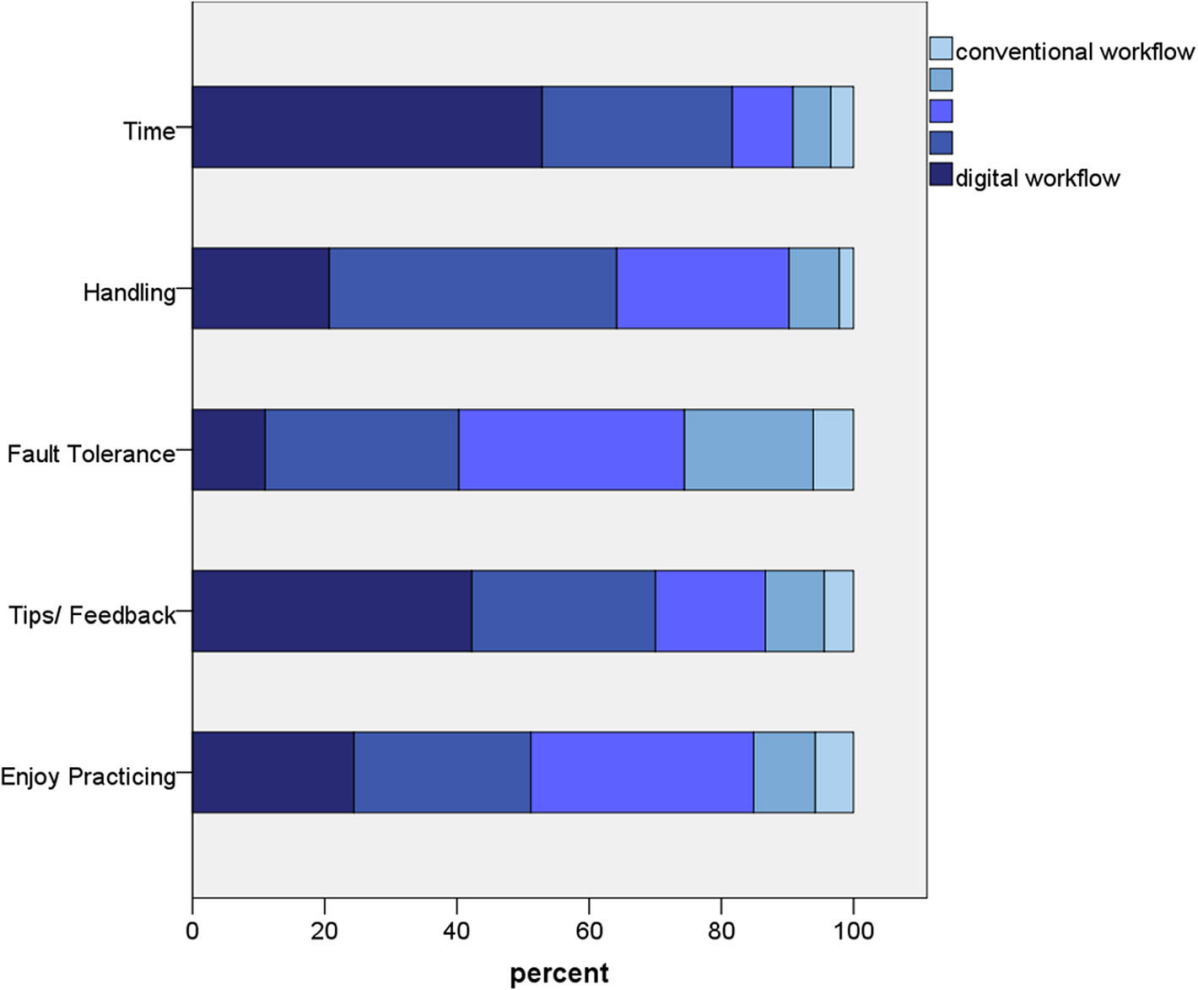
Depiction of the results of the questionnaire II – CAD/CAM-workflow regarding the aspects of using a conventional or digital workflow for manufacturing of FDPs

In general, students assessed the aspects of handling, didactic benefit, and motivation positively in both the questionnaires (Figs. 5 and 7, Tables 1 and 2). However, students from the training module I indicated difficulties with the scanning of the preparations (Table 1) and the understanding of the prepCheck report (Table 1). Furthermore, students stated that they preferred the evaluation of their preparations by the dentists instead of the software, especially regarding tips and feedback (Fig. 6, Table 1). Nevertheless, 86.2% of the students could imagine using the digital preparation analysis in their dental office in future.

**Table 1:** Items and descriptive statistics of the questionnaire I (Digital preparation analysis)

Item	Item description	25th percentile	Median	75th percentile	N
Handling 1^a^	The menu guidance of the intraoral scanner and prepCheck-software is easily comprehensible.	4	4	5	95
Handling 2^a^ (rec.)	The scanning process with the intraoral scanner handpiece does not cause me any difficulties.	3	4	5	98
Handling 3^a^	The analysis of the preparation with prepCheck is feasible.	4	4	5	96
Handling 4^a^	After the demonstration and exercises in the trainings module, I feel confident to use prepCheck by myself.	4	4	5	97
Didactic benefit 1^a^	The workflow of the intraoral scanner and prepCheck software is clear and understandable.	4	4	5	96
Didactic benefit 2^a^	Seeing my preparation on the monitor and getting an objective analysis with prepCheck, trains my own judgement	4	5	5	98
Didactic benefit 3^a^	Using prepCheck is as well training of intraoral scanning.	4	4	5	98
Didactic benefit 4^a^	The prepCheck report is helpful to improve my own performance regarding preparation.	4	4	5	99
Didactic benefit 5^a^	The prepCheck report is easy to understand.	3	4	5	97
Motivation 1^a^	Using new technologies (e.g. prepCheck) motivates me.	4	4.5	5	98
Motivation 2^a^	Analyzing my own preparation with prepCheck motivates me.	4	4	5	96
Motivation 3^a^ (rec.)	I do not think that prepCheck as an additional course component is a chore.	3	4	5	94
Overall assessment ^b^	If you consider the following aspects of the assessment of your preparation, what type of assessment would you prefer (dentist or prepCheck)? (1 = dentist, 5 = prepCheck)				
Overall assessment 1^b^	less time	2	3	4	90
Overall assessment 2^b^	easier handling	2	3	4	88
Overall assessment 3^b^	greater fault tolerance	1	2	3	88
Overall assessment 4^b^	more tips/ better feedback	1	2	3	90
Overall assessment 5^b^	enjoy practicing more	2	3	3	92

N = number of valid answers (total: *N* = 104)

^a^type of answer: 1 = strongly disagree, 5 = strongly agree

^b^type of answer: 1 = evaluation by dentist, 5 = evaluation by prepCheck

rec: Handling 2 and Motivation 3 were recoded before the analysis of questionnaire, so that higher values also express a more positive assessment here

**Table 2:** Items and descriptive statistics of the questionnaire II (CAD/CAM-workflow)

Item	Item description	25th percentile	Median	75th percentile	N
Handling 1^a^	The menu guidance of the intraoral scanner is easily comprehensible.	4	4	5	93
Handling 2^a^ (rec.)	The scanning process with the intraoral scanner handpiece does not cause me any difficulties.	3	4	4	94
Handling 3^a^	The digital modelling of the restoration is good to perform.	3	4	5	95
Handling 4^a^	After the demonstration and exercises in the training module, I feel confident to perform the entire CAD/CAM workflow by myself.	4	4	5	94
Didactic benefit 1^a^	The workflow of the CAD/CAM workflow is clear and understandable.	4	4	5	94
Didactic benefit 2^a^	Seeing my preparation on the monitor trains my own judgement.	4	5	5	95
Didactic benefit 3^a^	By participating the training module, I acquired a good knowledge of the CAD/CAM workflow.	4	5	5	93
Motivation 1^a^	Using new technologies (e.g. intraoral scanning) motivates me.	4	5	5	91
Motivation 2^a^	The manufacturing of CAD/CAM restorations of my own preparation motivates me.	4	5	5	92
Motivation 3^a^ (rec.)	I do not think that learning the CDA/CAM-workflow as an additional course component is a chore.	4	5	5	92
Overall assessment ^b^	Which workflow do you prefer considering the following aspects? (1 = conventional, 5 = digital)				
Overall assessment 1^b^	less time	4	5	5	89
Overall assessment 2^b^	easier handling	3	4	4	92
Overall assessment 3^b^	greater fault tolerance	2	3	4	82
Overall assessment 4^b^	more tips/ better feedback	3	4	5	90
Overall assessment 5^b^	enjoy practicing more	3	4	4	86

N = number of valid answers (total: *N* = 97)

^a^type of answer: 1 = strongly disagree, 5 = strongly agree

^b^type of answer: 1 = conventional workflow, 5 = digital workflow,

rec: Handling 2 and Motivation 3 were recoded before the analysis of questionnaire, so that higher values also express a more positive assessment here

Students from the training module II also reported difficulties while using the intraoral scanning handpiece (Table 2). In addition, digital modelling of the restoration was sometimes challenging (Table 2). Most of the students stated that they preferred the digital workflow to the conventional manufacturing of FDPs with respect to the aspects of time consumption, more tips/better feedback, easier handling, more enjoyable practicing experience, and greater fault tolerance (Fig. 7, Table 2). Among the students from module II, 96.8% of the students could imagine using an intraoral scanner in daily practice in future.

## Discussion

In recent years, the requirements of modern education have changed. *Welk* et al. described that 83.5% of the dental students at the University of Tennessee (USA) expected to be taught using the CAL approaches [23]. *Murbay* et al. recommended that in the current era of digital technology, modern teaching methods such as simulation trainers should be integrated into dental education, as is the standard in other businesses such as aviation or automobile traffic [4]. However, *Margaryan* et al. [24] advised against a radical shift from conventional teaching methods to digital technologies, as demanded by *Prensky* [25].

The concept for implementation of digital dentistry into our preclinical curriculum consisted of two training modules that enabled the CAL systems to be integrated into the existing curriculum without completely replacing the conventional teaching methods. This approach allows a step-by-step implementation, as recommended by *Welk* et al. [23] Furthermore, with single training modules the curriculum is more flexible to meet the needs of the students. *Margaryan* et al. described the high relevance of involving students in teaching and respecting their preferences of teaching methods to achieve the highest possible educational success [24]. Therefore, the training modules of the present study were evaluated using a Likert scale, which is a standard procedure for surveys in the field of medicine [26–28].

After the training modules, most of the students stated that they felt confident of using the digital preparation analysis by themselves or of manufacturing a chairside restoration using the CAD/CAM workflow. These results showed that the structure of the curriculum, consisting of lectures, demonstrations, and training of practical skills in small groups, seems to be comprehensible for the students. However, such a curriculum involves high staff costs, which is consistent with the experiences of other authors [6, 19].

However, it is controversial whether the digital learning systems improve students’ self-assessment skills. *Wolgin* et al. stated that students often overestimate their own performance [5]. In the present study, students did not assess their own performance with and without the digital preparation analysis, as all students should benefit from the CAL approach. Nevertheless, to reflect on their own preparations and to train their self-assessment skills, students presented their preparation analysis reports to each other according to the sandwich principle [21].

Based on the results of the questionnaires, it can be concluded that the aspect of motivation was rated very positively. *Mays* et al. stated that students who were involved in teaching showed more responsibility for achieving their own learning success [8]. Therefore, instead of using the preparation by a faculty as the master preparation, [6] students’ best exam preparations were used in the present study. This should motivate the students and show them that it is possible to achieve preparations analogous to the master preparation with their level of education at the end of the preclinical course.

For effective education, a sufficient number of CAL approaches are required to enable students to learn digital dentistry, which is clearly an obstacle in the introduction of these techniques. In 2002, *Welk* et al. [29] started a survey of all German universities regarding the use of CAL learning systems in dental education. They revealed a positive attitude towards digital technologies with low implementation in the clinical education [29]. These findings were confirmed by a university survey conducted in 2010, where CAD/CAM technology was offered as an additional course component in student education at just one university in Germany [30].

Due to the prosthodontic nature of their course students evaluated only full crown preparations which is a clear limitation of this study. In the future, CAL approaches should also be implemented in restorative dentistry (i.e. inlay preparations) as described by *Wolgin* et al. [5] Furthermore, multi center approaches would be fruitful to better establish CAL methods in the undergraduate curriculum.

The results of the present study showed that most of the students could imagine using an intraoral scanner for patient treatment in the dental office in future. Studies have shown that students implement what they have learned during their studies in treatments performed in future [31]. Therefore, it should be the duty of the universities to impart theoretical knowledge and practical skills of digital dentistry to ensure modern treatment options for patients. However, this cannot be achieved in a cost-neutral way, especially with respect to the necessary investment costs and personnel costs.

## Conclusions

This study showed a positive perspective of students on the implementation of digital dentistry in the preclinical curriculum. However, difficulties with CAL systems were reported and most students preferred evaluation of preparation by dental instructors. Thus, CAL approaches cannot replace dental instructors but offer an additional teaching method besides the traditional teaching of manual skills.

## Data Availability

The datasets of this article are available from the corresponding author on reasonable request.

## Abbreviations

CAD/ CAM: Computer-aided design/ computer-aided manufacturing
CAI/ CAS/ CAL: Computer-aided instruction/ computer-aided simulation/ computer-aided learning
CNC: Computerized numerical control
FDP: Fixed dental prosthesis
Ph1/ Ph2: Course of dental prosthodontics I/ course of dental prosthodontics II
